# Impact of population based indoor residual spraying in combination with mass drug administration on malaria incidence and test positivity in a high transmission setting in north eastern Uganda

**DOI:** 10.1186/s12936-023-04799-6

**Published:** 2023-12-13

**Authors:** Mulebeke Ronald, Wanzira Humphrey, Yeka Adoke, Van Geertruyden Jean-Pierre

**Affiliations:** 1 Makerere University School of Public Health, Kampala, Uganda; 2 Global Health Institute, University of Antwerp, Antwerpen, Belgium

## Abstract

**Background:**

Mass drug administration (MDA) and indoor residual spraying (IRS) are potent malaria burden reduction tools. The impact of combining MDA and IRS is not well documented. We evaluated the impact of MDA + IRS compared to IRS alone at a high transmission site in Eastern Uganda.

**Methods:**

A quasi-experimental study was implemented in Toroma and Kapujan subcounties in north eastern Uganda. Both subcounties received four rounds of IRS using primiphos-methyl (Acttellic SC300) 6–8 months apart from December 2016 to December 2018. Eligible residents of Kapujan simultaneously received MDA using dihydroartemesinin-piperaquine (DHA-PQ). Health facility data was used to monitor malaria case incidence rate and test positivity rates.

**Results:**

In the MDA + IRS arm, malaria incidence dropped by 83% (IRR: 0·17 (0.16–0.18); p < 0.001) in children under 5 year and by 78% (IRR: 0·22 (0.22–0.23); p < 0.001) in persons aged ≥ 5 years from the pre-intervention to the intervention period. In the IRS arm malaria incidence dropped by 47% (IRR: 0.53 (0.51, 0.56); p < 0.001) in children under 5 years and by 71% 0.29 (0.28, 0.30); p < 0.001) in persons aged ≥ 5 years. A drastic drop occurred immediately after the intervention after which cases slowly increased in both arms. Malaria test positivity rate (TPR) dropped at a rate of 21 (p = 0.003) percentage points per 1000 persons in the MDA + IRS arm compared to the IRS arm. There was a mean decrease of 60 (p-value, 0.040) malaria cases among children under five years and a mean decrease in TPR of 16·16 (p-value, 0.001) in the MDA + IRS arm compared to IRS arm.

**Interpretation:**

MDA significantly reduced malaria burden among children < 5 years however the duration of this impact needs to be further investigated.

## Background

Despite scaling up malaria control interventions, Uganda has the third highest incidence of malaria globally [1]. Children under 5 years and pregnant women are the most affected groups. The 2018/19 Uganda Malaria Indicator Survey (UMIS), reported a malaria prevalence of 9% by microscopy among children under 5 years [2].

The 2014/2020 Uganda Malaria Reduction Strategic Plan (UMRSP) set ambitious targets to reduce malaria mortality rate to 1 per 100,000 population/year, reduce malaria cases from 150 to 30 confirmed cases per 1000 population/year and to reduce parasite carriage to less than 7% [3]. Several malaria control interventions are implemented in Uganda including case management, use of long lasting insecticidal nets (LLIN), Intermittent preventive treatment in pregnancy (IPTp) and indoor residual spraying (IRS) in targeted high risk districts, however coverage and uptake of interventions is low [4, 5].

Vector control and case management alone may not be adequate to reduce malaria transmission in all areas to pre-elimination levels [6]. Subsequently, the new WHO guidelines for malaria recommend use of MDA in treating asymptomatic individuals to reduce parasite reservoir and malaria burden [7]. MDA is an empiric administration of a therapeutic anti-malarial regimen to an entire population simultaneously. This may contribute to the reduction of a residual parasite prevalence of approximately 20–30% though with a high potential for reinitiating of endemic transmission [8].

MDA may play a big role in optimizing gains achieved by IRS when implemented concurrently to target the host reservoir of malaria parasites [9] and accelerate malaria reduction from high transmission to low transmission and/or from low transmission to pre-elimination. However, there is limited evidence on the additional impact of MDA when used in combination with IRS for malaria burden reduction [7].

Our group conducted a study in Katakwi district in Eastern Uganda to assess the impact of MDA + IRS compared to IRS alone on malaria prevalence. Results from the study published elsewhere showed that malaria prevalence was significantly reduced in areas that received MDA + IRS [10]. In this paper we assess the impact of MDA + IRS compared to IRS alone using routine health facility data.

## Methods

The methodology of the main study is described elsewhere [10]. Briefly the study was a quasi-experimental study conducted in three sub counties (Kapujan, Toroma and Magoro) in Katakwi district in northeastern Uganda. All three sub counties lie along the southeastern edge of Katakwi district, and border Lake Bisina, a fingerling of Lake Kyoga. Toroma and Kapujan received four rounds of IRS using primiphos-methyl (Acttellic SC300) 6–8 months apart from December 2016 to December 2018. All eligible residents of Kapujan subcounty simultaneously received MDA using dihydroartemesinin-piperaquine (DHA-PQ). Magoro sub county served as a control. Standard malaria control interventions were implemented in all sub counties and included universal bed net distribution in 2017, provision of bed nets at ante natal care (ANC), intermittent preventive treatment in pregnancy (ITPp) and case management. Routine health facility data was collected over the intervention period. Cross sectional malaria prevalence surveys were conducted after every round of the intervention. The analysis in this paper is focused on the areas that received the interventions namely Kapujan and Toroma.

### Outcome measures

The main outcome measure was malaria case incidence rates and test positivity rates computed from routine health facility data aggregated in the health management information system (HMIS) at facility level and the District Health Information Software (DHIS2). We utilized data in HMIS from July 2015 to May 2019 from all 5 health facilities in the study area namely Kapujan health III, Kokorio health center II and Damasiko Health centre II in Kapujan Subcounty and Toroma Health center IV and Akurao health center II in Toroma subcounty. The health facilities were supported to collect high quality data and they achieved over 90% reporting rate.

Malaria incidence rate was computed as reported malaria cases per 1000 population per month. Test positivity rate was defined as the proportion of malaria tests that were positive. We computed outcomes during the pre-intervention period from (July 2015 to November 2016 (17 months before study intervention) and the intervention period from December 2016 to May 2019 (30 months of intervention implementation).

### Statistical analysis

Stata 14 (College Station, Texas 77845 USA) was used to perform statistical analysis. Malaria incidence rate was computed for children under five and for persons aged 5 years and above. The test positivity rate was computed for the same age groups.

Segmented Interrupted time series analysis was performed to test the hypothesis that a combination of MDA and IRS would greatly accelerate the reduction of malaria incidence rate and test positivity rate as compared to IRS. Time series analysis was used to assess the trends in malaria incidence and test positivity rate from the pre intervention period, at the first point of the intervention and during the intervention period.

We compared the change in the burden of malaria between the two study arms from the pre intervention to the intervention period, at the first point (month) of the intervention (level of change) and during the intervention period (coefficient in trends). A positive difference indicates an increase in the indicator and a negative difference, a decrease in the indicator.

We used a step and Poisson model to show direction of change of the indicators and their statistical significance. This model corrects for autocorrelation and adjusts the change estimate for possible time trends of the indicator during the pre-intervention period and for a possible immediate drop or rise of the indicator following the start of intervention and for a time trend on the indicator during the intervention period.

A difference-in-difference (DID) analysis was performed to assess the impact of the different interventions on malaria case incidence rate and test positivity rate from the pre-intervention to the intervention period using a pooled mean. We computed the difference in means of the outcome measures within study arms and between study arms. The DID estimator of the intervention effect with corresponding p-value at 0·05 significance level and 95% confidence interval was determined.

### Ethical considerations

This study was registered with the Pan African Clinical Trial Registry on 11^th^ July 2018 (PACTR 201807166695568). Study approval was obtained from the National Council of Science and Technology (UNCST) and by the Makerere University School of Public Health Higher Degrees Research and Ethics Committee (MUSPH-HDREC). Written consent was sought from the head of households or their designate to participate in IRS. Written consent was sought from heads of households or their designate for the children under their care to participate in MDA. All adults provided written consent to participate in MDA. Children aged 8–17 years provided written assent to participate in the study.

## Results

### Study flow

**Fig. 1:**
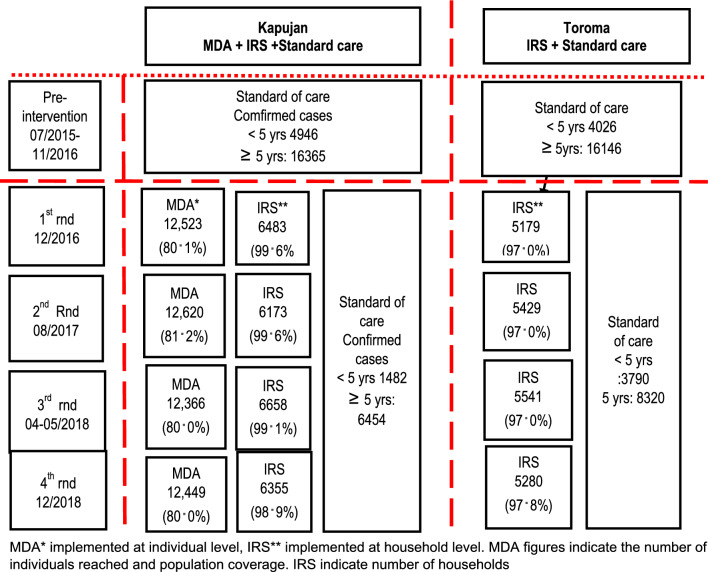
Study profile of additional public health implementations with MDA and/or IRS on top of standard of care in 2 sub counties in SE Uganda from July 2015- December 2018 MDA* implemented at individual level, IRS** implemented at household level. MDA figures indicate the number of individuals reached and population coverage. IRS indicate number of households

Interventions were implemented at population level involving all household for IRS and all eligible individuals for MDA. A total of four rounds of interventions were implemented between December 2016 and December 2018. Round 1 was conducted in 12/2016, round 2 in 08/2017, round 3 in 04–05/2018 and round 4 in 12/2018. In Kapujan subcounty MDA coverage was 80.1% in round 1, 81.2% in round 2, 80.0% in round 3 and 80.0% in round 4. IRS coverage was 99.6% in round 1, 99.6% in round 2, 99.1% in round 3 and 98.9% in round 4. In Toroma sub county, IRS coverage was 97.0% in round 1, 97.0% in round 2, 97.0% in round 3 and 97.8% in round 4. Details of the study areas, the interventions implemented in each area and the number of malaria cases before and during the intervention period are presented in Fig. 1.

### Baseline characteristics

The baseline sex and age distribution of the study participants are comparable across the study arms. Females constituted slightly over 50% of the population. Over 70% of the study population were aged 5 years and above. The study was conducted in a rural area with an average household size of 5.3. The baseline malaria incident rate during the pre-intervention period among children under 5 years and in those aged 5 years and above was 341.8/1000 population and 1131.1/1000 population in Kapjan subcounty and 340.5/1000 population and 1365.4/1000 population in Toroma subcounty respectively (Table 1). Baseline test positivity rate was 61% among children under five years and 47% among individuals aged five years and above in Kapujan subcounty while in Toroma it was 53% among children under five years and 47% among those aged five years and above. (Table 2).

**Table 1:** Changes in Malaria incidence rate (per 1000 population/month) in 2 sub counties in SE Uganda from July 2015 to May 2019

Study arm	Crude estimates		Incidence rate per 1000 population/month		Incidence rate difference 95% CI	Incidence rate ratio 95% CI	p-value
	Pre	During	Pre	During			
MDA + IRS							
< 5 years	4946	1482	6.70	1.13	−5.60 (−5.57, −5.37)	0.17 (0.16–0.18)	< 0.001
≥ 5 years	16365	6454	22.18	4.96	−17.22 (−17.58, −16.86)	0.22 (0.22–0.23)	< 0.001
IRS							
< 5 years	4026	3790	6.68	3.56	−3.11 (−3.35, −2.88)	0.53 (0.51, 0.56)	< 0.001
≥ 5 years	16146	8320	26.77	7.82	−18.96 (−19.40, −18.51)	0.29 (0.28, 0.30)	< 0.001

**Table 2:** Changes in Test Positivity Rate (TPR) in 2 sub counties in SE Uganda from July 2015 to May 2019

	Crude estimates		Change in TPR		Difference in change in TPR 95% CI	Test positivity ratio 95% CI	p-value
	Pre	During	Pre	During			
MDA + IRS							
< 5 years	61	22	0.08	0.02	−0.07 (−0.09, −0.04)	0.20 (0.12, 0.34)	< 0.001
≥ 5 years	47	22	0.06	0.02	−0.05 (−0.07, −0.03)	0.27 (0.15, 0.45)	< 0.001
IRS							
< 5 years	53	30	0.09	0.03	−0.06 (−0.09, −0.03)	0.32 (0.19, 0.51)	< 0.001
≥ 5 years	47	28	0.08	0.03	−0.05 (−0.08, −0.03)	0.34 (0.20, 0.55)	< 0.001

### Trends of malaria incidence and test positivity rates in children under five years

The malaria incidence and test positivity rates generally reduced from the pre-intervention to the intervention period across all study arms. In the MDA + IRS arm, malaria incidence rate reduced from 6.70/1000 population/month in the pre-intervention period to 1.13/1000 population/month in the intervention period. Malaria incidence dropped by 83% (IRR: 0.17 (0.16–0.18); p < 0.001). In the IRS arm, malaria incidence rate reduced from 6·68/1000 population/month to 3.58/1000 population/month dropping by 47% (IRR: 0.53 (0.51, 0.56); p < 0·001) (Table 1).

In the MDA + IRS arm, malaria test positivity rate (TPR) reduced from 0.08 in the pre-intervention period to 0·02 in the intervention period with a reduction of 80% (IRR; −0.20,CI 0.12, −0.34) while in the IRS arm, TPR reduced from 0.09 to 0.03, 68%(IRR; −0.32, CI −0.19, −0.51) (Table 2).

**Fig. 2:**
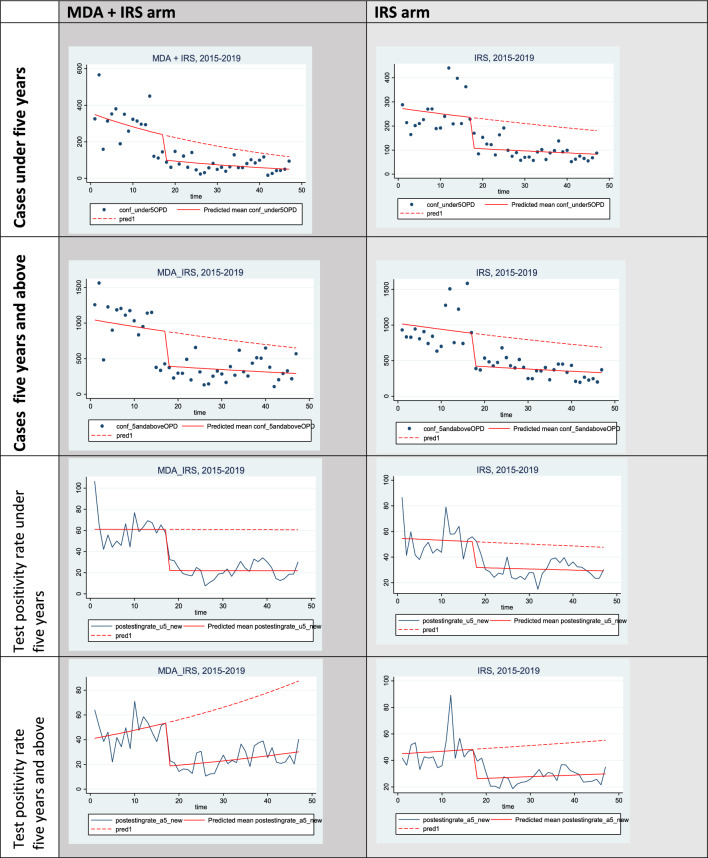
Comparing pre intervention and during intervention observed and predicted trends in malaria cases and Test Positivity Rates by study arm

There was a significant drop in malaria incidence of 72 cases per 1000 (p < 0.001) in the first month following MDA + IRS compared to the IRS arm. In the subsequent months of the intervention period, malaria incidence rate slowly increased at 6.85 cases/1000 persons per month (p = 0.002) in the MDA + IRS arm compared to the IRS arm, however the levels remained below the pre-intervention levels (Table 3, Fig. 2).

**Table 3:** Comparing the coefficients of malaria incidence rates between MDA + IRS and IRS arms among under five years and above five years from July 2015 to May 2019

Malaria incidence rate	< 5 years		≥ 5 years	
	Coefficient (95% CI)	P-value	Coefficient (95% CI)	P-value
Malaria incidence rate at beginning of study	84.47 (64.96, 103.99)	< 0.001	69.99 (59.63, 80.35)	< 0.001
Pre intervention trend of malaria incidence rate from July 2015 to November 2016	2.31 (0.28, 4.33)	0.026	1.82 (0.47, 3.17)	0.009
Reduction in malaria incidence rates at month 18 on implementing MDA + IRS compered to IRS	−71.82 (−96.56, −47.09)	< 0.001	−56.73 (−76.11, −37.35)	< 0.001
Trend in malaria incidence rate after implementing intervention:	−3.23 (−5.·24, −1.23)	0.002	−2.56 (−4.00, −1.12)	0.001
Difference in malaria incidence rate during interventions between MDA + IRS and IRS arms months after initiation of the interventions	6.85 (2.53, 11.19)	0.002	6.02 (3.07, 8.98)	< 0.001

There was similarly a significant drop in malaria TRP in the first month of the intervention of 21 percentage points per 1000 persons (p = 0.003) in the MDA + IRS compared to the IRS arm. In the subsequent months of the intervention period, there was a non-significant monthly increase in TPR at a rate of 0.29 percentage points per 1000 persons (p = 0.83) in the MDA + IRS arm compared to the IRS arm (Table 4, Fig. 2) however the levels remained below the pre-intervention levels.

**Table 4:** Comparing the coefficients of TPR between MDA + IRS and IRS arms among under five years and above five years from July 2015 to May 2019

Test positivity rate				
Test positivity rate at beginning of study	53.47 (38.36, 68.57)	< 0.001	41.22 (34.78, 47.66)	< 0.001
Pre intervention trend of Test Positivity Rate from July 2015 to November 2016	−0.02 (−1.41, 1.38)	0.978	0.68 (−0.16, 1.52)	0.11
Reduction in test positivity rates at month 18 on implementing MDA + IRS and IRS	−20.97 (−34.62, −7.31)	0.003	−25.33 (−39.67, −10.98)	0.001
Trend in test positivity rate after interventions	−0.09 (−1.57, 1.37)	0.897	−0.65 (−1.56, 0.26)	0.16
Difference in test positivity rate during interventions between MDA + IRS and IRS arms months after initiation of the interventions	0.29 (−0.29, 2.87)	0.825	0.76 (−0.76, 2.28)	0.32

### Trends of malaria incidence and test positivity rates in persons aged five years and above

The malaria incidence and test positivity rates generally reduced from the pre-intervention to the intervention period across all study arms. In the MDA + IRS arm, malaria incidence rate reduced from 22.18/1000 population/month to 4·96/1000 population/month. Malaria incidence dropped by 78% (IRR; 0.22, CI 0.22, 0.23). In the IRS arm, malaria incidence rate reduced from 26.77/1000 population/month to 7.82/1000 population/month. Malaria incidence dropped by 71% (IRR; 0.29, CI 0.28, 0.30). (Table 1).

In the first month following initiation of interventions, there was a significant drop in malaria incidence of 56.73 cases per 1000(p < 0.001) in the MDA + IRS arm compared to the IRS arm. In the subsequent months of the intervention, malaria cases slowly increased at a monthly rate of 6.02 cases per 1000 persons (p < 0.001) in the MDA + IRS arm compared to the IRS arm (Table 1), however the levels remained below the pre-intervention levels.

TPR generally reduced from the pre-intervention to the intervention period across all study arms. In the MDA + IRS arm, TPR reduced by 73% from 0.06 to 0.02, (IRR; −0.27, CI 0.15, 0.45) while in the IRS arm TPR reduced by 73% from 0.08 to 0.03, (IRR; −0.27, CI 0.15, 0.45) (Table 2).

In the first month following the intervention, TPR dropped at a rate of 25.33 per 1000 persons (p = 0·001) in the MDA + IRS compared to the IRS arm. There was however, a non-significant monthly increase in TPR in the subsequent months of the intervention at a rate of 0.76 percentage points per 1000 persons (p = 0.32) in the MDA + IRS arm compared to the IRS arm, however the levels remained below the pre-intervention levels (Table 4, Fig. 2).

### Comparison of difference-in-difference (DID) of change in malaria incidence and test positivity rate from pre-intervention to intervention period within and between the study arms

The mean number of malaria cases reduced from the pre-intervention to the intervention period in all study arms. The mean number of malaria cases in children under five years significantly reduced by 60 (p-value, 0.04) in the MDA + IRS compared to the IRS alone arm. In persons aged 5 years and above, the mean number of malaria cases reduced by 48 (p-value, 0.62) in the MDA + IRS compared to the IRS alone arm.

The mean TPR in the MDA + IRS arm significantly decreased by 16 percentage points (p-value, 0.001) among children under five in the MDA + IRS compared to the IRS alone arm. However, among those aged five years and above, we observed a non-significant reduction in the mean TPR (mean reduction = 4.23; p-value, 0.309). (Table 5).

**Table 5:** Difference in mean percentage of malaria cases and test positivity rates in IRS + MDA and IRS arm by age

Outcome variable	Pre—intervention 17 months			During—intervention 30 months			Difference-in-difference (DID) Estimator (P-values)
	MDA + IRS N(Mean)	IRS N(Mean)	Diff (MDA + IRS)—(IRS)	MDA + IRS N(Mean)	IRS N(Mean)	Diff (MDA + IRS)—(IRS)	(MDA + IRS) Vs (IRS)
			Mean (p-value*)			Mean (p-value*)	
Conf-OPD							
Under 5	4946 (291)	4315 (254)	37.12 (0.11)	1482 (72)	2048 (75)	−22.80 (0.19)	−**60 (0**.**040)**
Over 5	16365 (963)	16146 (950)	12.88 (0.87)	6254 (341)	8320 (375)	−34.60 (0.55)	−48 (0.617)
Overall	2311 (1254)	20,461 (1204)	50.0 (0.60)	7736 (413)	10368 (470)	−57.40 (0.42)	−107.40 (0.368)
TPR							
Under 5	60	53	7.7 (0.04)	22	30	−8.48 (0.003)	−**16**.**16 (0**.001*)
Over 5	47	47	0.35 (0.92)	24	27	−3.88 (0.124)	−4.23 (0.309)
Overall	50	48	1.88 (0.54)	24	28	−4.69 (0.047)	−6.57 (0.093)

Bold value is the key result discussed in this paper

* represents statistical significance

## Discussion

We assessed the impact of MDA + IRS compared to IRS alone on malaria burden using routine health facility data. The findings showed that IRS with or without MDA had immediate significant reduction on malaria incidence rates and TPR, however this gradually wanes with time. In the MDA + IRS arm, and IRS arm, malaria incidence rate significantly reduced by 83% and 47% in children under five years and by 78% and 71% in those aged five years and above respectively.

Malaria test positivity rate (TPR) reduced by 80% in the MDA + IRS arm and by 68% in the IRS arm in children under five years. In persons aged 5 years and above, TPR reduced by 73% and 66% in the MDA + IRS arm and IRS arm respectively.

There was a significant drop in malaria incidence and TPR in the first month following MDA + IRS compared to the IRS arm in all age groups. After the first month of the intervention, malaria incidence rate and TPR slowly increased in the MDA + IRS arm compared to the IRS arm, however the levels remained below the pre-intervention levels. The mean number of malaria cases and mean TPR significantly reduced from the pre-intervention to the intervention period in the MDA + IRS compared to the IRS alone arm in children under five years compared to individuals 5 year and above. We discuss the added impact of MDA observed among children under 5 years compared to individuals five years and above. The added impact of MDA in young children may be attributed to vulnerability to high numbers of parasite in their blood due to low immunity [11] compared to older individuals hence associated with higher incidence of malaria [12]. Age, exposure and immunity determine the varying levels of malaria parasitemia among individuals. The repertoire of immune responses increases with age and active infection which is experienced more among adults [13]. Although other studies show varying success of MDA [14, 15], findings from this study suggest MDA had added impact on malaria incidence particularly among younger children. As observed in other studies, MDA can be used to reduce and interrupt transmission in specific settings [16, 17] notably where transmission is low and moving toward malaria elimination [18]. In this study the decline in malaria incidence rate and TPR reveals impact on malaria transmission [19]. Numerous factors may be associated with the observed changes in malaria incidence rate and TPR in children under five years; such as difference in baseline malaria cases, difference in trends over the study period, proportion of patients tested, care-seeking behavior and utilization of health facilities.

Our findings further show immediate effects of MDA on malaria parasites influencing TPR directly while IRS acts indirectly through killing mosquitoes and effects are seen after weeks following interventions (Fig. 2). This suggests that MDA augments the effect of IRS on malaria burden. This finding is supported by modelling studies which have shown how adding rounds of MDA increases the effect of vector control interventions [20, 21]. MDA rapidly reduced malaria incidence rate suggesting that targeting both the vector and the parasite is a plausible approach [18, 22] necessary if malaria control efforts are focused towards pre-elimination. Given findings from this study, MDA may accelerate vector control impact, aiding effort toward elimination in high transmission settings.

This study further shows that the added value of MDA was realized following the first round of interventions. Notably because MDA will accelerate elimination by reducing the starting number of infections as observed elsewhere [23], the challenge is to keep transmission low especially in malaria endemic settings where re-infection is high. The observed small overall effect and rising monthly cases and TPR following the month of interventions could be due to the wide implementation interval of 6–8 months apart between interventions yet dihydroartemesinin-piperaquine used for MDA has a post treatment prophylactic protective period of approximately 1 month [24]. This suggest that MDA may be more appropriate to be administrated monthly. Similar reasons could explain why there were minimal differences between intervention groups on malaria incidence rates in this study, though there are varying results of MDA impact on malaria incidence rates elsewhere [25, 26].

### Study limitations

Results of this study should be interpreted in light with some limitations. comparing treatment and control group was used to generate meaningful trends. Secondly, malaria incidence rate at the beginning of the study and the baseline trend were similar in both arms but at different levels. However, the long period of monthly trends showed comparable trends in the two study arms. In addition, routine health facility surveillance data has limitations for example access to care although this could be similar in the study area. Issues of consistence and completeness which may arise affect the assessment of trends of disease occurrence although widely used in Sub-saharan Africa to assess impact of malaria control interventions [27, 28]. Finally, TPR included both malaria rapid diagnostic tests (mRDT) and/ or microscopy tests done on patients presenting at the out-patient department. TPR could be an over estimation however, this is unlikely to change results in trends as it was assumed that this error was applied across the duration of study period in the study arms, therefore the trends are more likely to be true of what happened in the study area.

### Generalisability of study findings

MDA is a potential vital tool in interrupting malaria transmission and is worth considering as a potential key strategy for malaria control and elimination in settings with high malaria intensity. Indeed, the added value of MDA on IRS in our study is minimal but can be generalized to other similar settings in the context of accelerating reduction of malaria burden from high to low transmission intensities especially among children under 5 years.

## Conclusion

Our study shows that the combination of MDA and IRS reduced more malaria incidence cases and test positivity rate in addition to LLINs, and case management among children under 5 years than in 5 years and older age individuals during the study period from December 2016 to December 2018. The use of IRS and ITNs may be sufficient in reducing malaria burden in all age, however, in areas with high transmission intensity, reducing morbidity will likely require a combination of both a wide coverage of a community chemoprevention such as MDA in addition to IRS and LLINs. Robust designs such as randomized controlled trials to evaluate impact of MDA and IRS on malaria burden in high transmission settings may be required to provide evidence on the sequence in which malaria control intervention are layered to optimize levels of impact and maximize chances of sustained control or successful elimination. Both MDA and IRS may be an appropriate mix of interventions in accelerating reduction and sustain low transmission as countries navigate from high transmission to low transmission and from low transmission to pre-elimination.

## Data Availability

Dataset used for this manuscript are available and are uploaded.

## Abbreviations

DID: Difference-in-difference
IRS: Indoor residual spraying
ITS: Interrupted time series
LLIN: Long lasting insecticide treated net
MDA: Mass drug administration
MTR: Mid term review
MOH: Ministry of health
SPH-HDREC: School of Public Health Higher Degrees Research & Ethics Committee
UNCST: Uganda National Council of Science & Technology
UNMCP: Uganda National Malaria Control Programme
UMRSP: Uganda Malaria Reduction Strategic Plan
WHO: World Health Organization
